# Continuous non-contact monitoring of neonatal activity

**DOI:** 10.1186/s12887-024-05238-4

**Published:** 2025-02-25

**Authors:** Paul S. Addison, Dale Gerstmann, Jeffrey Clemmer, Rena Nelson, Mridula Gunturi, Dean Montgomery, Sam Ajizian

**Affiliations:** 1 Research and Development, Acute Care and Monitoring, Medtronic, The Technopole Centre, Edinburgh, UK; 2 Timpanogos Regional Hospital, Orem, UT USA; 3 Clinical Research and Medical Science, Acute Care and Monitoring, Medtronic, Boulder, CO USA

**Keywords:** Patient monitoring, Neonatal ICU, Machine learning, Random forest algorithms, Motion

## Abstract

**Purpose:**

Neonatal activity is an important physiological parameter in the neonatal intensive care unit (NICU). The degree of neonatal activity is associated with under and over-sedation and may also indicate the onset of disease. Activity may also cause motion noise on physiological signals leading to false readings of important parameters such as heart rate, respiratory rate or oxygen saturation or, in extreme cases, a failure to calculate the parameter at all. Here we report on a novel neonatal activity monitoring technology we have developed using a Random Forest machine learning algorithm trained on features extracted from a depth video stream from a commercially available depth sensing camera.

**Methods:**

A cohort of twenty neonates took part in the study where depth information was acquired from various camera locations above and to the side of each neonate. Depth data were processed to provide features indicating changes corresponding to the activity of the neonate and then input into a Random Forest model which was trained and tested using a leave-one-out cross validation paradigm.

**Results:**

Applying the thresholds found in training the Random Forest model during testing with leave-one-out cross validation, the mean (standard deviation) of the sensitivity and specificity of the optimal points and the corresponding area under the receiver operator curve (ROC-AUC) were 92.0% (8.8%), 93.2% (11.1%) and 97.7% (2.5%) respectively. The activity identified by the model also appeared to match well with noisy segments on the corresponding respiratory flow signal.

**Conclusions:**

The results reported here indicate the viability of continuous non-contact monitoring of neonatal activity using a depth sensing camera system.

**Supplementary Information:**

The online version contains supplementary material available at 10.1186/s12887-024-05238-4.

## Introduction

Neonatal activity, in the form of gross body movement or the more localised movement of limbs, hands, head, etc., is an important physiological parameter in the NICU. The degree of neonatal activity may be associated with under- and over-sedation, seizures or the lethargic response to disease. Pain is a significant cause of increased activity in neonates and may be a consequence of diagnostic or therapeutic interventions, or directly through a disease process. Effective management of pain involves the administration of appropriate sedative and analgesic drugs in order to mitigate against the manifestation of long-lasting physiologic or neurodevelopmental consequences [1]. Underactivity, in the form of lethargy, has been found to be the most frequent vital sign associated with the onset of sepsis [2]. In addition, regardless of the root cause, activity in the form of limb motion or gross movement of the torso can also introduce significant motion noise to physiological signals used for monitoring in the NICU [3]. This may lead to erroneous reporting of vital signs such as respiratory rate and associated apnea alarms, heart rate and saturation levels. These two major considerations of neonatal activity are drivers of our current research concerning the development of a robust and continuous, non-contact neonatal activity monitor.

**Fig. 1:**
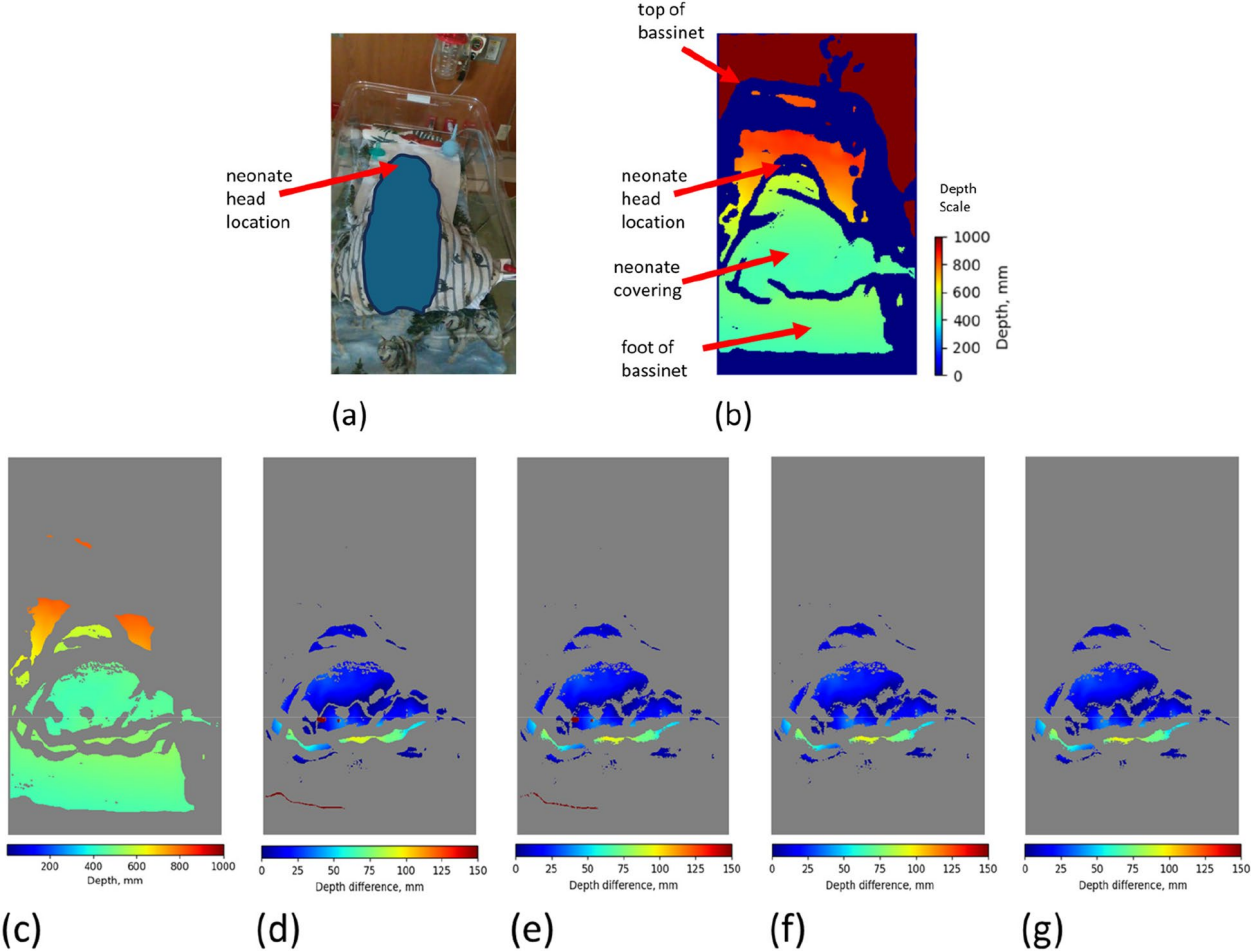
A Screen Capture from the Display showing the Neonate Depth Image and Associated RGB Image together with subsequent Processing Steps to find Depth Differences. **a** RGB image – note that all identifying feaures of the neonate have been blanked out from the image. **b** Median averaged depth frame from Intel D415 camera video stream (note that the camera is placed at the foot’s end of the bed and therefore distances are closer near the bottom of the image). **c** Median averaged depth frame after removing depths > 87th percentile and < 3rd percentile. **d** Depth difference frame, calculated by subtracting consecutive median depth frames. **e** 5 × 5 and 3 × 3 spatial median filters applied. **f** Depth differences > 150 mm removed (too large to be neonatal motion). **g** Regions with area < 40 pixels removed. (The depth difference scales for (**d**) to (**g**) are also shown in the figure.)

Depth cameras are emerging as a powerful new tool that can provide a continuous measure of information without the need to attach a sensor to the patient. These include respiratory rate and tidal volume [4–9], respiratory patterns [10, 11] and apneas [12–14]. These cameras provide a matrix of distances (‘depths’) to all objects within the field of view. We may visualize what the depth camera is sensing by rendering the matrix of depths. An example is provided in Fig. 1 which contains a color rendered depth image of a neonate lying in an open bassinet (Fig. 1(b)) with the corresponding standard color RGB image for comparison (Fig. 1(a)). The distance scale is also provided for the depth image. We hypothesized that changes in depth associated with gross body movements could provide the basis for the non-contact ‘touchless’ monitoring of neonatal activity. This would target larger scale body motions not associated with the smaller scale motions corresponding to respiratory activity. Here we report on this work, where we compare the detected activity with a manually scored truth label. During the study we had the opportunity to investigate the effect on performance of various bed types, coverings and camera locations.

## Methods

### Data acquisition and processing

We collected depth data from 20 neonates in the NICU (Wasatch Neonatal, Timpanogos Regional Hospital, Orem, UT). Institutional Review Board (IRB)- approved informed consent, including consent to publish, was obtained for each participant covering the essential information stated in the protocol, as required elements according to 21 CFR 812.150 for a non-significant risk medical device investigation, and in accordance with the guidelines proposed in the Declaration of Helsinki. The neonates were situated in isolettes (*N* = 9) and bassinets (*N* = 11) as appropriate for their condition and were not on ventilatory support. Demographic data for the neonates is provided in Table 1.

**Table 1:**
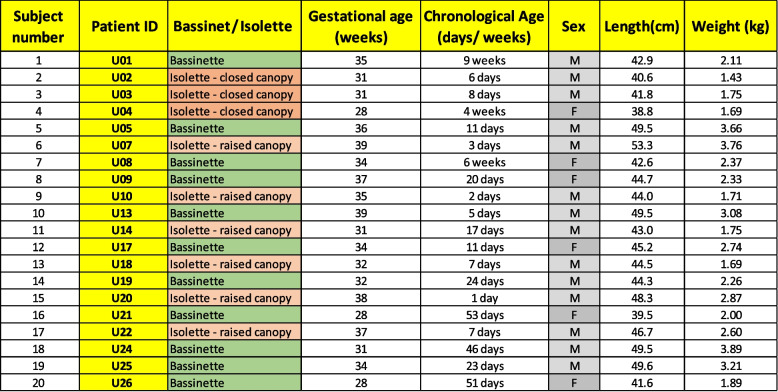
Demographic data of the neonates participating in the study

Depth information was acquired from the scene using an Intel RealSense™ D415 camera connected to a laptop at a frame rate of 30 fps. The camera was mounted on a tripod and placed at different positions around each neonate. Distances ranged from 230 to 800 mm between the camera and neonate’s chest. Various camera locations were used relative to the isolette or bassinet (above, above-side, side, foot of the bed, head of the bed). The camera was placed at six different positions for 17 neonates. For two neonates, the camera arm malfunctioned during the depth data collection, hence, depth data for only 3 and 5 positions, respectively, were collected, and for one further neonate, depth data was inadvertently collected for only 5 positions. Thus, a total of 115 positional test-run acquisitions were conducted for the 20 neonates. In total, over 35,000 s of test data were acquired and analysed.

The depth data was acquired using our depth camera system which provides a real-time streaming display of the rendered depth image of the neonate. This is a modification of a system that was originally developed by our group detecting respiratory activity in adult patients [5, 9].

We developed a binary classifier to monitor neonatal activity from the depth camera video stream. This was achieved by converting the depth video stream into “depth difference” frames by computing the changes in depth between frames at 1-s intervals and implementing denoising steps. Figure 1(a) shows the RGB image capture of a neonate and Fig. 1(b) shows the corresponding temporally-averaged depth capture where the camera was placed at the foot of the bed. Temporally-averaged depth captures were obtained by dividing the depth video stream into 1-s spans and finding the median average of the 10 frames at the start and end of every 1-s span. By examining these temporal averages, we may detect motion in the scene related to activity as changes in depths over time. This is achieved by first pre-processing the depth averages to remove noise prior to extracting features to use in the model. These are described as follows:

Figure 1(c) to (g) indicate the noise removal steps that were implemented to exclude depth changes outside the frame region where the neonate is located. Firstly, depths higher than the 87th percentile, and depths lower than the 3rd percentile, of all depths in the range 200 mm – 1000 mm are removed from the frame (Fig. 1(c)). The percentiles were found through a grid search which was used to optimize model performance. The distances between the camera and the neonate are within the range 230 mm – 800 mm; thus, suitable percentile limits within the range of 200 mm – 1000 mm were determined and applied to include only depths pertaining to the neonate’s location in the frame. Next, the depth video stream was converted into depth difference frames by computing the changes in valid depths between consecutive depth frames at 1-s intervals (Fig. 1(d)). Two spatial median filters of kernel size 5 × 5 and 3 × 3 were then applied to the depth difference frames, to remove small pixel regions containing unusually high or low depth values and thus smooth out the regions of motion (Fig. 1(e)). To ensure that only depth differences corresponding to neonatal motion were included in the frame, high depth difference values of > 150 mm were removed from the frame (Fig. 1(f)). Finally, small regions of valid depth differences (with area < 40 pixels) were removed, since these were considered too small to be valid neonatal motion (Fig. 1(g)). An example video sequence of the depth difference values over time can be found in the Supplementary material associated with this manuscript. Photographs of examples of the open isolette, closed isolette and bassinet used in the trial are also provided in the Supplementary material.

**Fig. 2:**
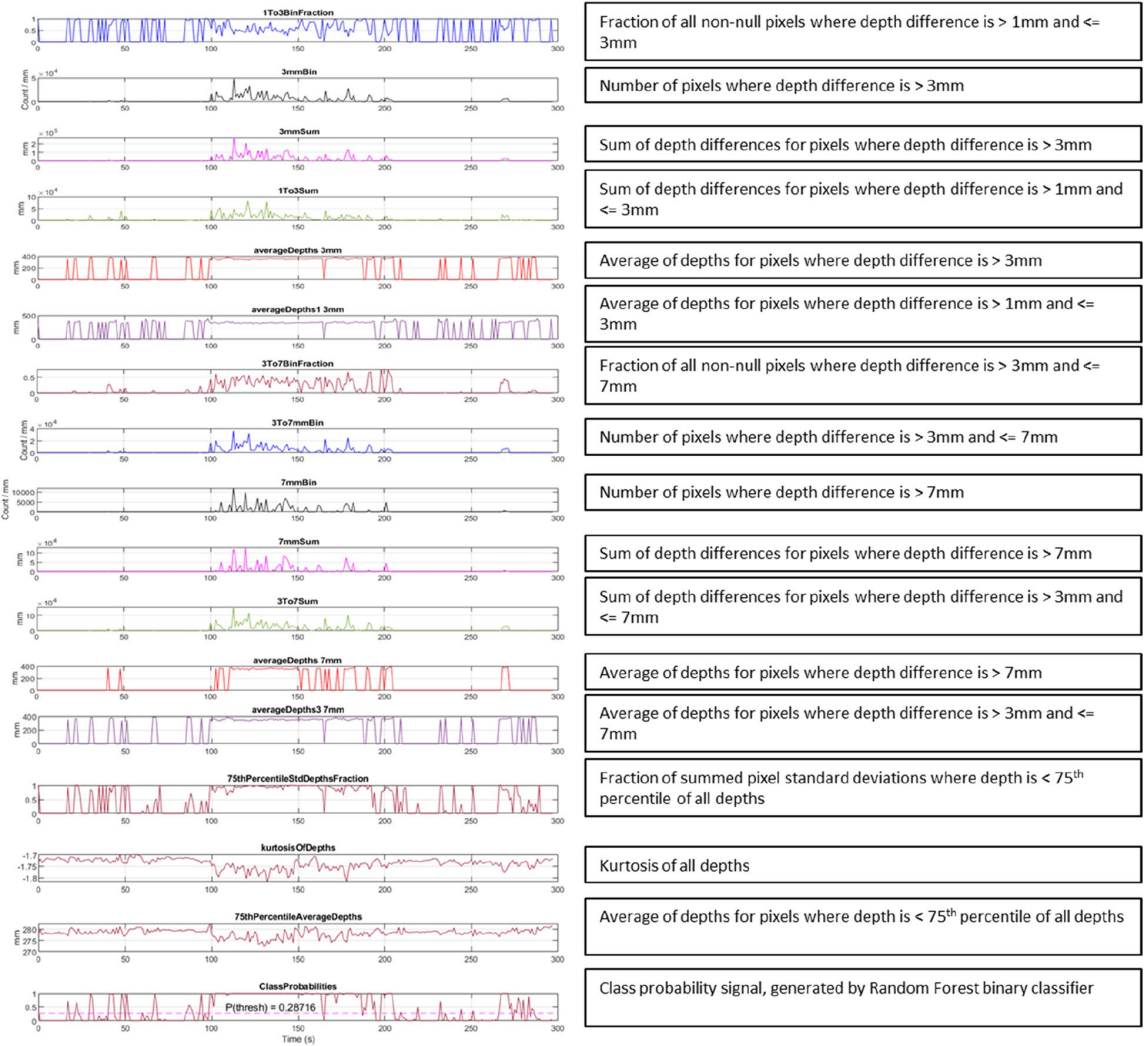
A time series of the 16 input features from an example depth video stream with the resulting class probability signal shown at the bottom

After implementation of the denoising steps, sixteen time-variant features were extracted from the depth and depth difference information from the depth video and used to train the model: a Random Forest classifier. Figure 2 contains example plots of the time-variant features. The features were chosen to represent small and large motions that occur over varying spatial areas and are normalized based on the average of valid depths within the frame. The sixteen features may be grouped as follows: Five depth difference features were designed to represent the spatial scale of motion, to detect motions of different depth changes that occur over a larger spatial area. Four summed depth difference features were designed to represent the magnitude of motion, for the detection of motions of various magnitudes over a small spatial area. Four average depth features were designed to normalize the depth difference features with respect to distance from the camera, enabling detection of motions corresponding to a wide range of depth changes. This allows the classifier to learn different neonatal movements. Three final features were designed to detect motion from depth streams where the camera was placed at a skewed angle (head’s end or foot’s end). A 75th-percentile depths feature was designed to capture the pixel standard deviations. A kurtosis of depths feature represents the amount of skew in the depths distribution and is thus indicative of the amount of noise in the frame; this helps to minimize false positives when classifying noisy depth streams. Finally, a 75th-percentile depths feature was designed to filter out depths higher than the 75th percentile, corresponding to noise, and provide a measure of how far away the scene is from the camera. This provides information to the model to allow it to normalize according to distance.

### The random forest classifier

**Fig. 3:**
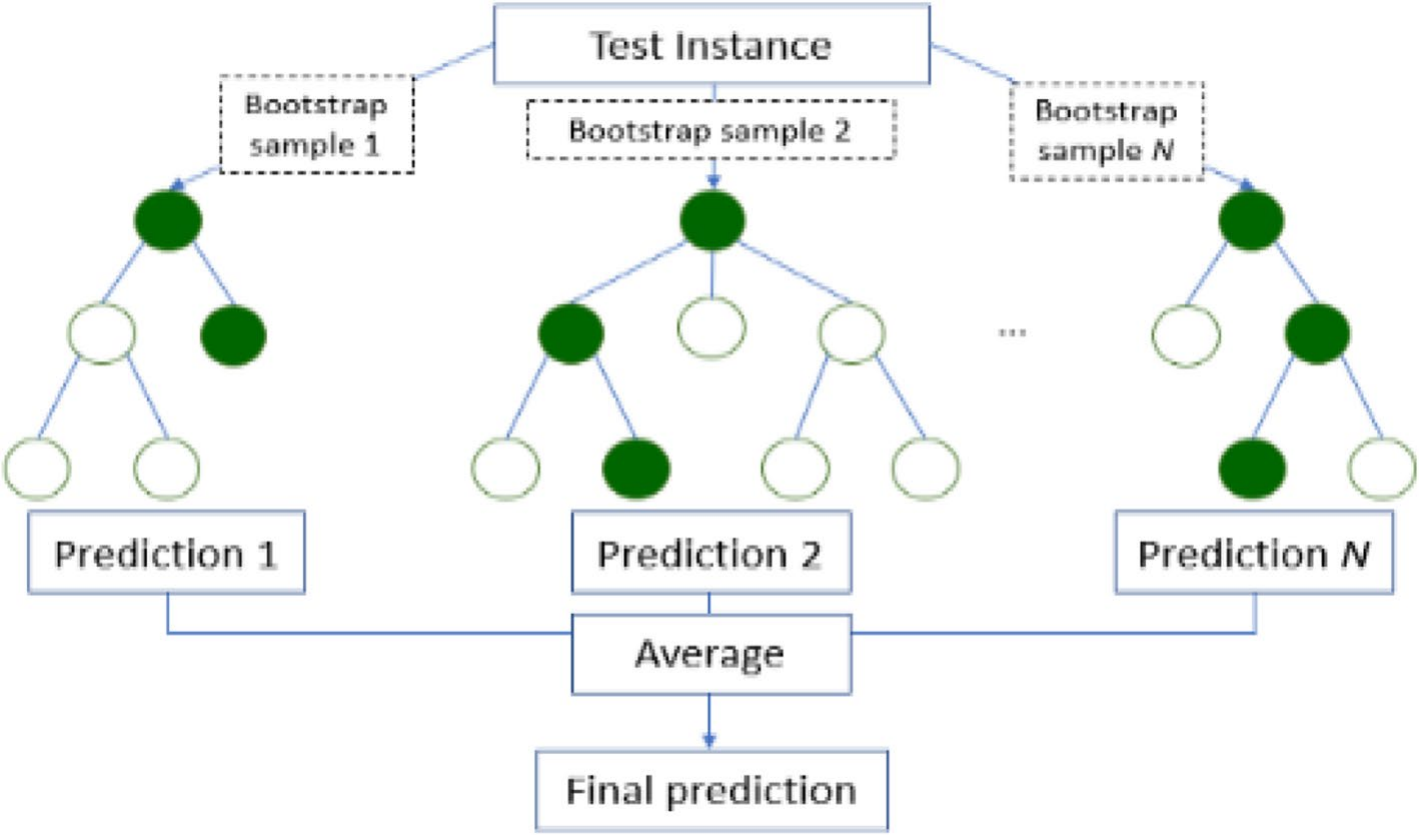
Random forest classifier architecture

The machine learning model implemented here is a binary Random Forest classifier that predicts “motion” or “no motion” for each second of the video stream. The Random Forest model is an ensemble of individual Decision Trees [15], each trained on a bootstrap sample (a sample drawn with replacement) of the data; a random set of features is used to train each tree. These factors increase the diversity of the model [16]. To calculate the final class probability (probability of there being motion or no motion in a given second) of the model, the class probabilities of all the trees in the Random Forest are averaged- this minimizes overfitting in the model [17]. A schematic of the Random Forest classifier is provided in Fig. 3.

Optimization of the Random Forest model hyperparameters (e.g. the number of trees in the ensemble, maximum number of features used in training, maximum tree depth) and the denoising parameters (e.g. number of temporally-averaged frames, spatial median filter size, depth percentile limits) was based on a grid-search approach that aimed to maximize the average square root of the resulting test ROC curve AUCs (see next section). This was to minimize both the number of false positives and false negatives in the predicted motion signal.

### Data analysis

The classifier was trained and tested on a manually scored motion truth signal determined from visual observation of the corresponding RGB video stream. This was performed by one of the authors (MG), where each RGB video sequence was assessed to determine whether any detectable motion was present in the scene. This was achieved by splitting the video into 1-s frames, then viewing the frames sequentially to determine if visually observable neonatal motion was present between frames. This amounted to approximately 35,000 s of labelled video data. The data for each neonate consists of up to six depth video captures, each containing around 300 s of video recorded under different capture conditions and variables including:

- [1] Bed type: bassinet, covered isolette, uncovered isolette.
- [2] Cover type: bare skin, sleep suit, loosely covered, loosely swaddled, tightly swaddled, nested.
- [3] Camera location: above, above-side, foots end, heads end, side.
- [4] Distances from the camera to the neonate (center of chest): ranging from 230 to 800 mm.

A leave-one-out cross validation (per-neonate) approach was used for the analysis where labelled data from 19 neonates was used to train the model. This was then tested on the data from the remaining subject for each of the individual positional data sets (i.e. for each camera location). This was repeated 20 times, such that the model was tested on each of the positional test runs from all 20 neonates, until all 115 positional test data sets were analysed.

A ROC curve analysis was used, where a training ROC curve with corresponding class probability threshold points was first determined for each of the aggregated 19 neonatal data sets. The optimal class probability threshold for the remaining (‘one-left-out’) data set was selected by choosing the threshold at which the mean of the sensitivity and specificity of the training results is maximized.

We also investigated the effect on the performance of the capture conditions as described above. The ROC AUC was computed for each sub-category and results were further split according to the individual neonate.

## Results

**Fig. 4:**
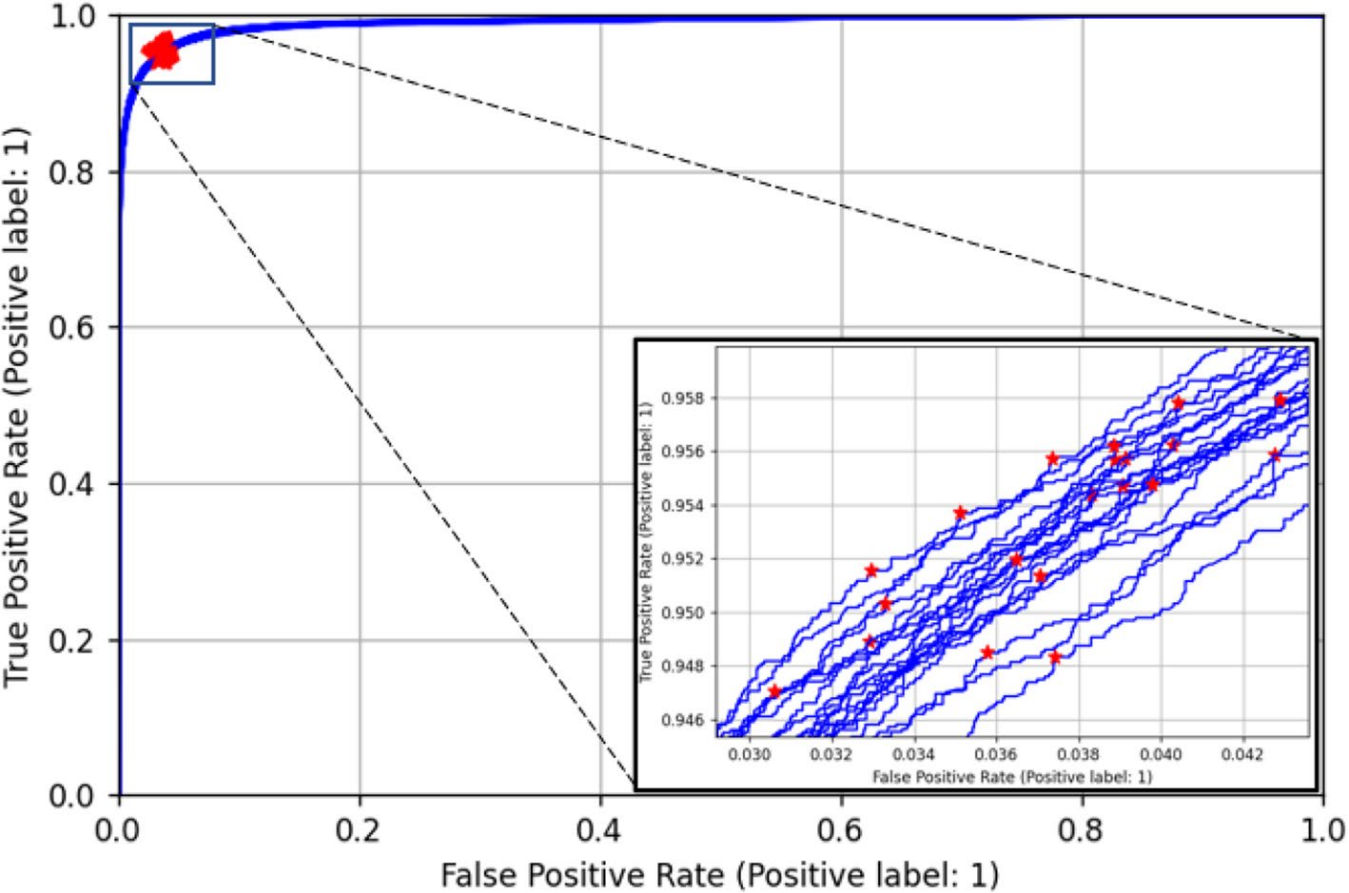
Training ROC curves

**Fig. 5:**
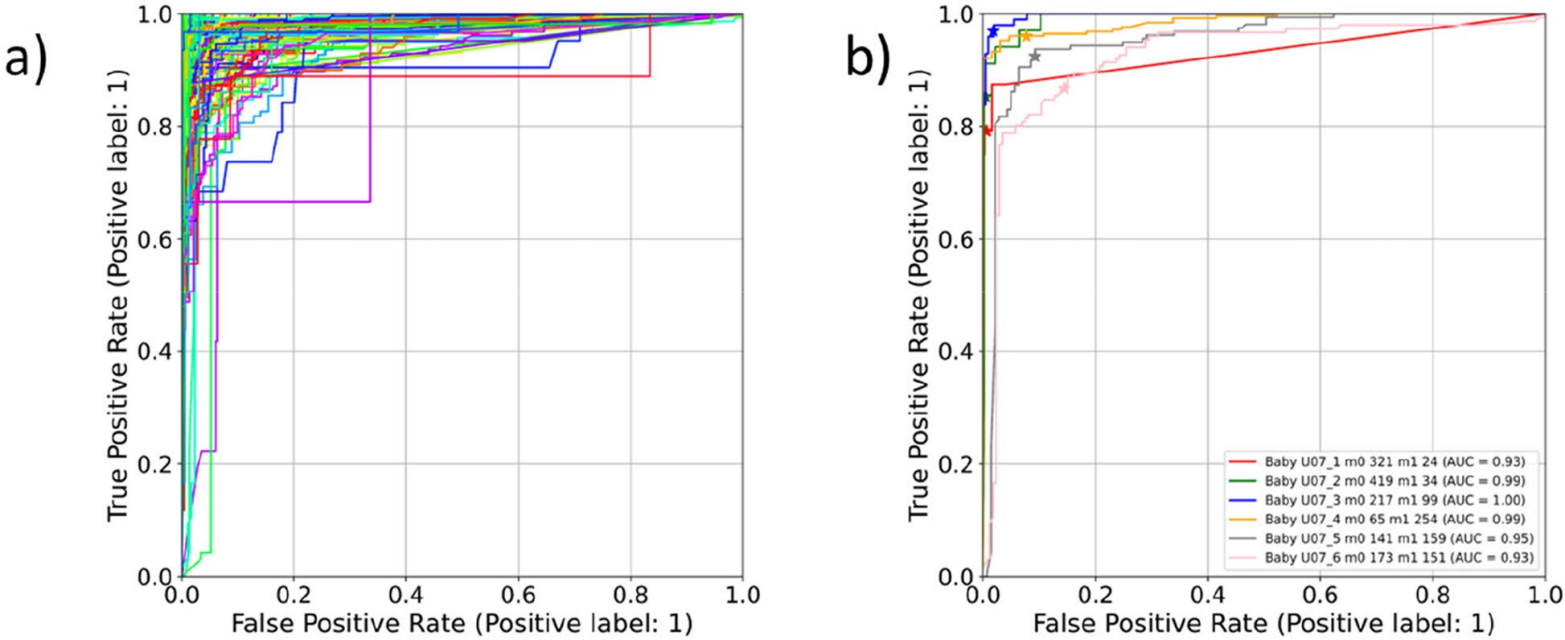
Test ROC curves. **a** All 115 Test ROC Curves. **b** Example of 6 Test ROC curves for Neonate 7 showing the location of the sensitivity/specificity pairs obtained from the applied threshold for this neonate

Figure 4 shows the 20 training ROC curves with the optimal points indicated for each of the 20 training runs. We can see that the curves and optimal points on the curves are similar, as each aggregated set differs only by the remaining ‘left-out’ set. The class probability threshold corresponding to the optimal ROC curve point for each run was then determined. The position of the optimal points found on each ROC curve are shown in the zoom in plot in Fig. 4. The mean (standard deviation) of the sensitivity and specificity of the optimal points, the corresponding ROC-AUC and the resulting thresholds are 95.3% (0.3%), 96.3% (0.3%), 99.0% (0.0%) and 0.272 (0.020) respectively. The thresholds generated through this training process were then applied to the corresponding test data for each set of positional data from the remaining neonate. Using each threshold, the model was then applied at each second of depth video data to classify motion in the scene (defined as when the predicted final class probability is greater than the threshold). The ROC curves for all 115 depth clips tested in this way are shown in Fig. 5(a). An example of the ROC curves for a subset of this plot is shown in Fig. 5(b) with the sensitivity/specificity pairs resulting from the applied threshold marked. We can see a much larger variability in the results than for the training set (as we would expect). The mean (and standard deviation) of the sensitivity and specificity of the optimal points and the corresponding ROC-AUC for the test sets using the thresholds found in the training are 92.0% (8.8%), 93.2% (11.1%) and 97.7% (2.5%) respectively.

**Fig. 6:**
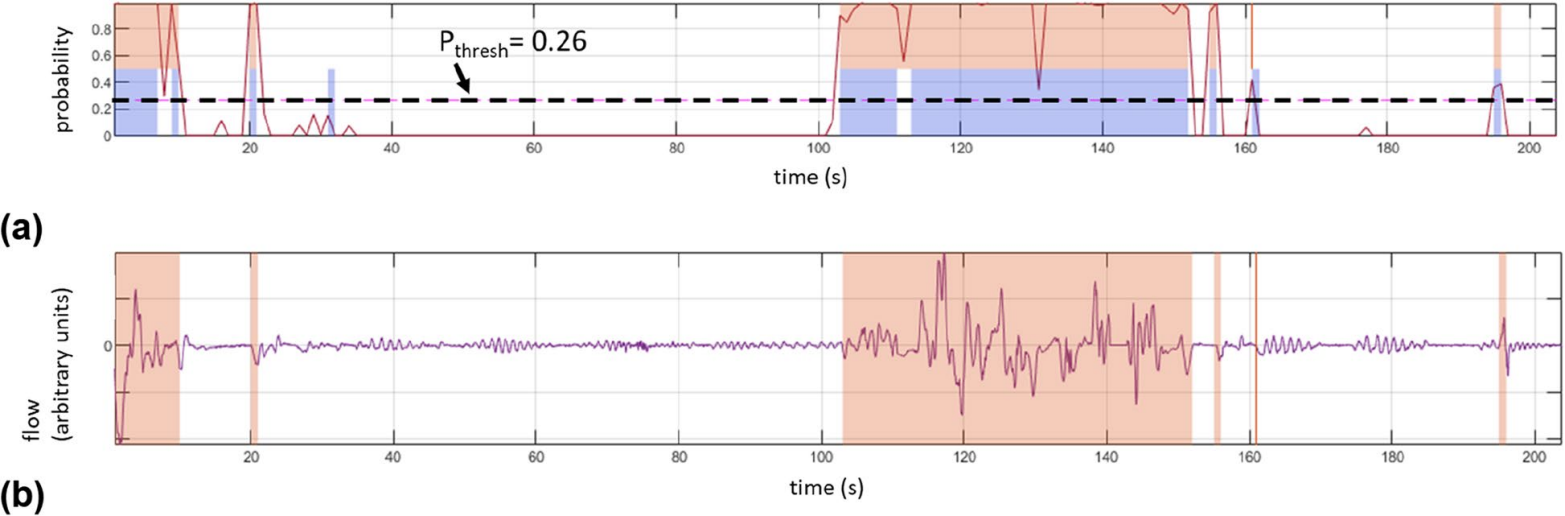
Model Results. **a** The class probability signal with the predicted motion and observed motion (truth) signals is indicated by red and blue patches respectively. **b** The respiratory flow signal with the predicted activity regions indicated

Figure 6 (a) illustrates a typical example result of the model. Periods of predicted motion and the motion truth signal are indicated by red and blue respectively on the class probability signal. The class probability threshold for this neonate (P_thresh_) is indicated on the plot by the horizontal dashed line. Figure 6(b) shows an example of the predicted motion flag superimposed on the respiratory flow signal derived from the depth camera by integrating respiratory depth changes at the chest region of the neonate. (The calculation of a respiratory flow signal using the depth system focuses on the much smaller respiratory motions in the scene. This is not the focus of the work described here and is more fully described in [4, 9]). The patches match well with the large-scale fluctuations in the signal which correspond to observed motion of the neonate.

**Fig. 7:**
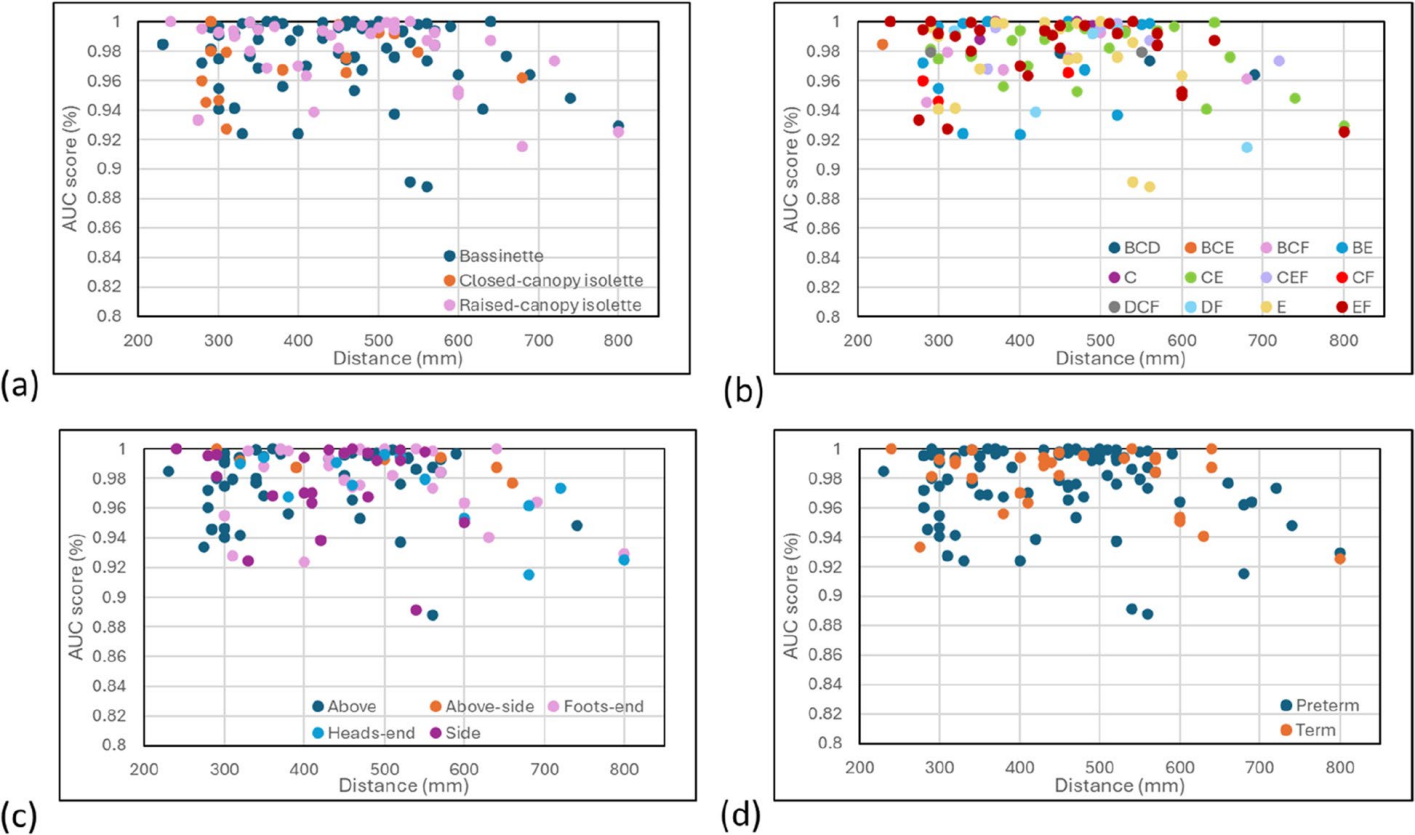
Individual ROC AUC Scores split according to distance from the neonate and (**a**) bed type, **b** covering type, **c** camera location, **d** term/preterm split. For (**b**) the covering legend is: A: bare skin. B: Sleep suit. C: Loosely covered. D: Loosely swaddled. E: Tightly Swaddled. F. Nested

We further investigated the effect of bed type, covering type and camera location on the results corresponding to each neonate. Figure 7(a), (b) and (c) contains the results from these analyses. In addition, at the suggestion of a reviewer, we performed a split in terms of term/preterm age (> 37 weeks / ≤ 37 weeks), plotted in Fig. 7(d). We discerned no obvious pattern in the ROC AUC performance for any of these parameters. However, viewed as a whole (i.e., looking only at the AUC score versus distance from any of the four plots and viewing the top right portion of the data points) there does appear to be a steadily increasing drop off in performance at or around, 640 mm to 800 mm.

## Discussion

A major cause of increased activity in neonates is pain caused by diagnostic or therapeutic interventions, or directly through a disease process. Pain management involves the administration of appropriate sedative and analgesic drugs in order to mitigate against the manifestation of long-lasting physiologic or neurodevelopmental consequences [1]. However, correct titration of the drug requires an assessment of pain where the clinical evaluation of both over- and undersedation is required. A wide variety of manual pain and sedation scales are available [2] including the Neonatal Pain, Agitation and Sedation Scale (N-PASS) [18, 19]. N-PASS includes the manual assessment of activity as part of the score to determine both lethargy, associated with oversedation, or restlessness, associated with undersedation and pain. In addition to oversedation, neonatal lethargy may be a result of hypoxic-ischemic encephalopathy, infection/sepsis, asphyxiation, inborn errors of metabolism, intraventricular hemorrhage, or hyperbilirubinemia [20]. Underactivity, in the form of lethargy, has been found to be the most frequent vital sign associated with the onset of sepsis when considered among a range of other vital signals including grunting, abdominal distension, increased prefeed aspirates, tachycardia, hyperthermia, chest retractions [21]. An improved prediction of late-onset sepsis in the NICU was achieved by Cabrera-Quiros et al. in their machine learning model by including body movement information [22]. Both studies highlight the importance of this underactive state in predicting disease. Neonatal seizures are another potential generator of pathological activity including convulsive movements [23]. Seizures may originate from a range of causes including hypoxic ischemic encephalopathy, infarction or intracranial hemorrhage, intracranial infections, brain malformations, genetic or metabolic disorders. The degree of general body movements in neonates has also been associated with apnea events, where it can indicate the maturity of the neuro-respiratory system [24]. In other work by the same group it was demonstrated that the durations of movement bouts increases with age in the preterm infant [25].

Regardless of the source, activity in the form of limb motion or gross movement of the torso can also introduce significant motion noise to physiological signals collected in the NICU. The affected signals generally include the transthoracic impedance (TTI) signal, the electrocardiogram (ECG), and the photoplethysmogram (PPG), which are used to monitor respiratory rate and apnea, heart rate and saturation levels. These form the principal modalities for ABD (apnea-bradycardia-desaturation) monitoring in the NICU [26, 27]. Motion artifact often contaminates all three of these signals [3] and is a challenge for robust, accurate and continuous neonatal monitoring. According to Jorge et al. [28], the high prevalence of noise and high false alarm rates is the reason that respiratory signals are still largely disregarded in neonatal intensive care units. In addition, it is sometimes difficult to distinguish between motion artefacts and cardiac (pulsatile) signal in the PPG when the frequency of motion is close to the frequency of the heart rate [29]. Significant motion may cause the acquisition of stable and accurate PPGs to be delayed, especially during critical situations where, occasionally, the signal may even fail to appear [30]. These observations of the effect of motion on physiological signals resonate with our own group’s experience in working with a wide variety of biosignals. Both within the adult and neonatal domain, motion interference may lead to erroneous readings or a failure to calculate the number at all.

The neonatal activity algorithm that we have developed could form the basis of technology for the improved monitoring of the neonate, both by providing a quantifiable measure of activity and as an indicator of motion noise which could be used to improve vital sign monitoring. Developed using a Random Forest binary classifier to detect motion from a depth video stream, our activity algorithm achieved excellent performance when tested against labelled data on a leave-out-one cross validation protocol. The results indicate that the model can detect neonatal movements of varying magnitude and spatial scale. In addition, the classified segments appeared to match well with regions of respiratory flow containing significant noise caused by motion of the neonate. There was no obvious effect on the results from bed types, covering types or camera position, nor was there any discernible pattern when we performed a term/preterm split. It is likely that a considerably larger dataset would be required to tease out underlying relationships of this nature. However, it appeared from the limited data that the performance degraded when the camera was positioned at distances of over 640 mm from the neonate.

A limitation of the study was that it was conducted on a relatively small cohort of 20 neonates from a single NICU and, as such, should be taken as a pilot study. Future efforts will be aimed at labelling data from a larger cohort of neonates from multiple sites where, for example, relationships between the variables in Fig. 7 could be better discerned and also the relationship between activity and health status of the neonate could be studied. A major strength of the technology is that it only requires an off-the-shelf depth camera with no hardware changes. The system also requires no calibration, is simple to operate and does not require sensors to be attached to these most vulnerable of patients.

Video cameras are already commonplace in the NICU to enhance parent-infant attachment and reduce parental stress [31, 32]. As such, the NICU offers the potential for the seamless integration of a depth sensing camera system for neonatal activity monitoring. The camera used in the study reported here, the Intel RealSense™ D415, offers RGB, IR and depth modalities, and thus could be used to live stream color video images to the parent or caregiver while offering the depth modality for activity monitoring which could be streamed separately for clinical evaluation. Further, the depth camera system may offer opportunities for additional physiological monitoring in the NICU, such as respiratory rate, respiratory volume, respiratory patterns and apnea detection, as it has been shown to do so for adults [4].

## Conclusion

The results reported here demonstrate the viability of continuous non-contact monitoring of neonatal activity using a depth sensing camera system. Future work will seek to further improve the model by targeting a larger patient population at multiple sites and over longer monitoring periods.

## Supplementary Information

Supplementary Material 1.

Supplementary Material 2.

## Data Availability

Availability of data and materials: data is not available for commercial reasons. However, the results are easily replicated. The corresponding author may be contacted in this regard.
