# Supplementary material for: Continuous non-contact monitoring of neonatal activity
Source: BMC Pediatr. 2025 Feb 25;25:134. doi: 10.1186/s12887-024-05238-4 (PMC11853281; doi:10.1186/s12887-024-05238-4)
Supplement: Supplementary file 1 — Supplementary Material 1. [file 12887_2024_5238_MOESM1_ESM.pdf]

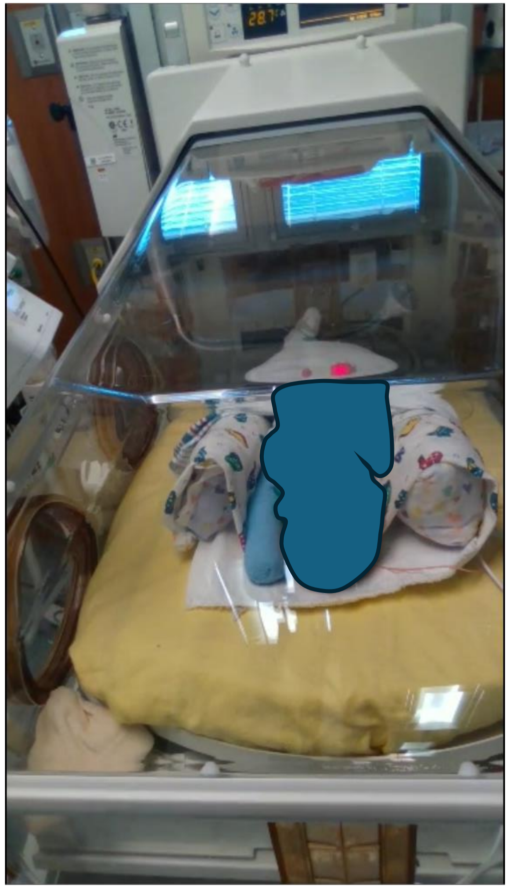

(a) U03 position 5, closed canopy isolette

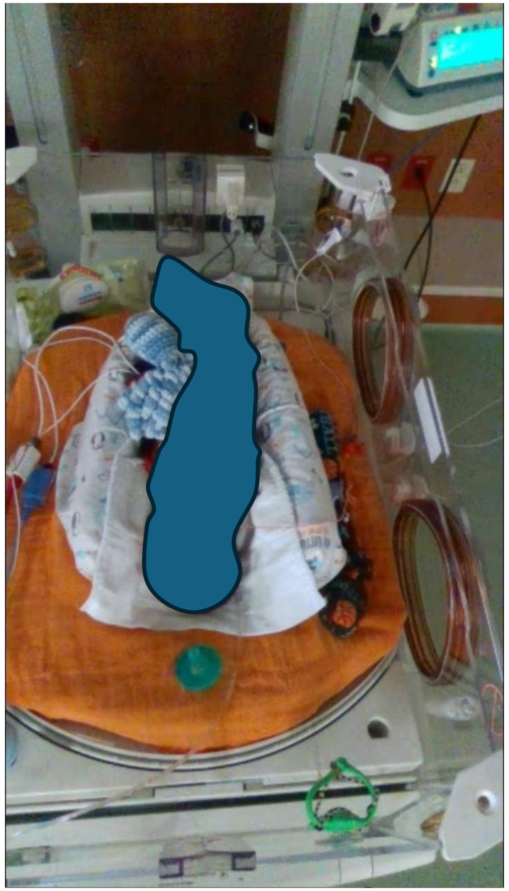

(b) U14 position 6, raised canopy isolette

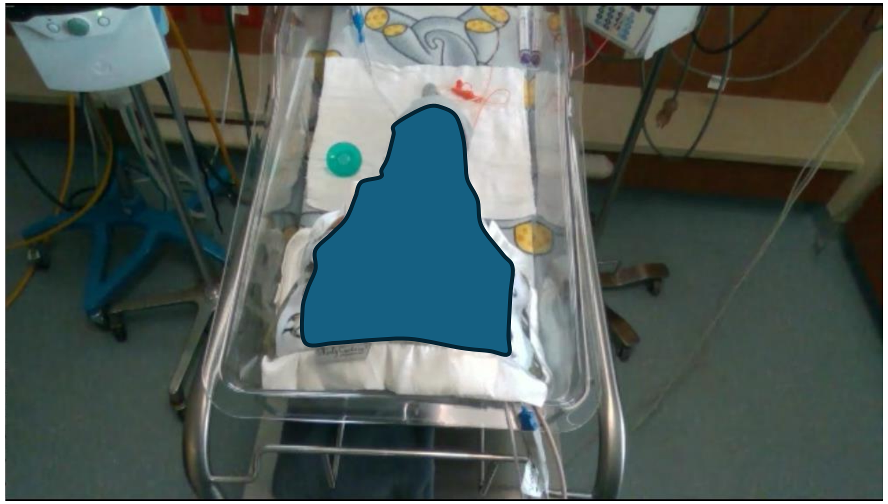

(c) U01 position 6, bassinet

Supplementary Figure: Example images of the three bed types & conditions

Note that all identifying features of the neonates have been blanked out from the images
